# The Effects of Tocilizumab on Inflammatory and Differentiation Pathways in Primary Human Chondrocytes: A Bioinformatic and In Vitro Approaches

**DOI:** 10.1111/1756-185X.70336

**Published:** 2025-07-09

**Authors:** Bugrahan Regaip Kilinc, Feyza Kostak, Omer Faruk Yilmaz, Suray Pehlivanoglu, Duygu Yasar Sirin

**Affiliations:** ^1^ Department of Biology, Institute of Natural and Applied Sciences Namık Kemal University Tekirdag Turkey; ^2^ Department of Molecular Biology and Genetics Necmettin Erbakan University Konya Turkey; ^3^ Corlu State Hospital Tekirdag Turkey

**Keywords:** bioinformatics, differentiation pathways, IL‐6 signaling, primary chondrocytes, Tocilizumab

## Abstract

**Introduction:**

This study investigates the effects of Tocilizumab, an interleukin‐6 (IL‐6) receptor inhibitor, on human primary chondrocyte cells, focusing on bone morphogenetic protein 2 (BMP‐2), hypoxia‐inducible factor 1‐alpha (HIF‐1α), interleukin‐1 beta (IL‐1β), SRY‐box transcription factor 9 (SOX‐9), and IL‐6 genes.

**Methods:**

Combining bioinformatic and experimental approaches, we assessed Tocilizumab's impact on inflammatory signaling pathways, cellular differentiation, and viability. Reactome and Gene Ontology (GO) enrichment analyses revealed the involvement of interleukin signaling, BMP, and mitogen‐activated protein kinase (MAPK) pathways.

**Results:**

Protein–protein interaction (PPI) network analysis indicated strong interactions among the studied genes, with BMP‐2 and SOX‐9 identified as central nodes. Western blot analysis demonstrated a 71% reduction in SOX‐9, a 55% reduction in HIF‐1α, and an 81% reduction in BMP‐2 expression levels by day 15. Conversely, IL‐1β levels decreased by 67% after prolonged treatment. MTT assays showed a 27.7% reduction in cell viability at day 15 compared to controls. Despite these changes, staining analyses confirmed preserved cell membrane integrity and nuclear morphology, indicating minimal cytotoxic effects.

**Conclusion:**

These findings highlight Tocilizumab's role in modulating inflammation and differentiation pathways in human primary chondrocytes. Further studies should explore the long‐term effects of IL‐6 blockade on cartilage remodeling and regenerative capacity in chronic inflammatory settings.

**Abbreviations**
ADAMTS: A Disintegrin and Metalloproteinase with Thrombospondin motifs
ANOVA: analysis of variance
AO/PI: acridine orange/propidium iodide
BMP‐2: bone morphogenetic protein 2
BP: biological process
CC: cellular component
COL2A1: collagen type II alpha 1 chain
DMEM: Dulbecco's Modified Eagle Medium
FBS: fetal bovine serum
GO: Gene Ontology
HBSS: Hank's balanced salt solution
HIF‐1α: hypoxia‐inducible factor 1‐alpha
IL‐1β: interleukin‐1 beta
IL‐6: interleukin‐6
MAPK: mitogen‐activated protein kinase
MF: molecular function
MMP13: matrix metalloproteinase‐13
MTT: 3‐(4,5‐dimethylthiazol‐2‐yl)‐2,5‐diphenyltetrazolium bromide
NF‐κB: nuclear factor kappa B
OA: osteoarthritis
PBS: phosphate‐buffered saline
PPI: protein–protein interaction
PVDF: polyvinylidene difluoride
RA: rheumatoid arthritis
RUNX2: Runt‐related transcription factor 2
SD: standard deviation
SOX‐9: SRY‐box transcription factor 9
STRING: Search Tool for the Retrieval of Interacting Genes/Proteins
TNMD: tenomodulin

## 1 Introduction

Cartilage tissue damage and inflammation are central features in the progression of various musculoskeletal disorders, including osteoarthritis (OA) and rheumatoid arthritis (RA) [1]. Chondrocytes, the primary cellular component (CC) of cartilage, play a crucial role in maintaining the structural and functional integrity of the cartilage matrix [2]; however, inflammatory cytokines can disrupt this balance, leading to cartilage degradation and loss of function [3]. Among these cytokines, interleukin‐6 (IL‐6) is recognized as a key mediator of inflammation and joint degeneration, promoting catabolic activities that accelerate cartilage breakdown [4]. Consequently, targeting the IL‐6 signaling pathway has emerged as a promising therapeutic strategy for modulating inflammation and preserving cartilage health [5, 6].

Tocilizumab, a humanized monoclonal antibody targeting the IL‐6 receptor, has shown clinical efficacy in reducing inflammation and inhibiting IL‐6‐mediated pathways [7, 8]. While its effects on systemic inflammatory diseases such as RA are well‐documented, the specific molecular mechanisms by which Tocilizumab influences chondrocyte functionality remain less explored. Understanding these mechanisms is essential for optimizing therapeutic strategies for cartilage preservation in inflammatory diseases. Although Tocilizumab has proven systemic anti‐inflammatory effects, little is known about its direct impact on cartilage‐resident chondrocytes at the molecular and cellular levels. Understanding these mechanisms is essential for optimizing therapeutic strategies for cartilage preservation in inflammatory diseases.

This study focuses on four key genes: bone morphogenetic protein 2 (BMP‐2), hypoxia‐inducible factor 1‐alpha (HIF‐1α), IL‐1β, SRY‐box transcription factor 9 (SOX‐9), and IL‐6 due to their critical roles in chondrocyte function, inflammation, and cellular stress response. BMP‐2 is integral to cartilage development and repair [9], while HIF‐1α regulates cellular adaptation to hypoxic conditions commonly observed in damaged cartilage [10]. SOX‐9 is a master regulator of chondrocyte differentiation and cartilage matrix synthesis [11, 12]. BMP‐2 promotes SOX‐9 expression, and HIF‐1α can further regulate SOX‐9 under hypoxic conditions, suggesting cross‐regulatory interactions among these factors critical for cartilage homeostasis [6]. Interleukin‐1 beta (IL‐1β) is a potent pro‐inflammatory cytokine that plays a key role in cartilage degradation by promoting catabolic processes and inhibiting extracellular matrix synthesis [13]. In addition, IL‐6 is not only a major inflammatory cytokine but also directly impacts chondrocyte metabolism and cartilage degradation [14, 15].

To elucidate the regulatory effects of Tocilizumab on these key pathways, we employed a combination of bioinformatic and experimental approaches. Reactome and Gene Ontology (GO) enrichment analyses were utilized to identify pathways significantly impacted by Tocilizumab treatment, while protein–protein interaction (PPI) network analysis provided insight into the interconnected roles of these genes. Complementary in vitro analyses, including MTT assays and staining analyses for cell viability and Western blot for protein expression, further explored Tocilizumab's effects on chondrocyte functionality. This study aims to provide a comprehensive understanding of Tocilizumab's role in modulating inflammation and chondrocyte survival, contributing to the development of targeted therapies for cartilage‐related inflammatory diseases.

## 2 Materials and Methods

### 2.1 Data Collection and Gene Selection

In this study, we examined the regulatory effects of Tocilizumab on human primary chondrocytes by focusing on four key genes: BMP‐2, HIF‐1α, IL‐1β, SOX‐9, and IL‐6. These genes were selected due to their critical roles in inflammation, cartilage degradation, and stress response, which are integral to chondrocyte functionality and the progression of cartilage‐related diseases. To facilitate pathway enrichment analysis, gene symbols were converted into Entrez IDs using the bitr() function from the R package clusterProfiler, with the human genome annotation database org.Hs.eg.db serving as the reference [16].

### 2.2 Functional Enrichment Analysis

To investigate the regulatory pathways influenced by Tocilizumab in human primary chondrocytes, Reactome pathway enrichment analysis and GO enrichment analysis were performed. Reactome pathway analysis was conducted using the enrichPathway() function from the ReactomePA package in R, with a statistical significance threshold of *p* < 0.05. The analysis utilized the org.Hs.eg.db database for gene annotation, and significant pathways were visualized using ggplot2. For functional classification, GO enrichment analysis was performed using the enrichGO() function from the clusterProfiler package. The selected genes were categorized into three principal domains: biological process (BP), molecular function (MF), and CC. GO terms with *p*‐values < 0.05 were considered statistically significant, and the results were visualized in a combined dot plot. All analyses were conducted in R (version 4.3.3.), and gene annotation was referenced against the org.Hs.eg.db database. Data preprocessing and normalization steps were performed prior to enrichment analysis to ensure the accuracy and reliability of pathway associations [16, 17].

### 2.3 Protein–Protein Interaction (PPI) Analyses

PPI networks were constructed to explore the broader interaction landscape of the identified genes, including BMP‐2, HIF‐1α, IL‐1β, SOX‐9, and IL‐6, in the context of Tocilizumab treatment. The Search Tool for the Retrieval of Interacting (STRING) database was utilized for the prediction and visualization of interaction networks, applying a confidence score threshold of > 0.4 to ensure the robustness of the analysis. The threshold of 0.4 (medium confidence) was selected to balance sensitivity and specificity, minimizing false‐positive interactions while capturing relevant biological associations. Key hub gene identification was directly performed using the Maximal Clique Centrality (MCC) algorithm in Cytoscape's CytoHubba plugin to determine the most critical proteins within the network. This approach provided insights into the molecular mechanisms potentially regulated by Tocilizumab treatment [18, 19].

### 2.4 Preparation of Human Primary Chondrocyte Cultures

Human primary chondrocyte cultures were established using cartilage tissue obtained from patients classified as Kellgren–Lawrence radiographic grade IV undergoing total knee arthroplasty (four males, four females; mean age: 44.12 ± 4.87 years). Tissues from patients with neutropenia, leukopenia, thrombocytopenia, active tuberculosis, or active hepatitis B or C were excluded from the preparation of primary cell cultures. Individuals with a history of liver or kidney dysfunction, pregnant women, and those with known allergies or hypersensitivity to Tocilizumab were not included in the study. Additionally, tissues from patients who received nonsteroidal anti‐inflammatory drugs, disease‐modifying antirheumatic drugs, or biological agents within the last month were also excluded from the present study. Tissues were transported aseptically in sterile Falcon tubes containing Dulbecco's Modified Eagle Medium (DMEM; cat no: 11965092, Thermo Fisher Scientific) supplemented with 5% penicillin–streptomycin (cat no: 15140122, Thermo Fisher Scientific). Following arrival, tissues were washed with phosphate‐buffered saline (PBS), mechanically dissociated, and digested overnight in Hank's balanced salt solution (HBSS; cat no: 14170070, Thermo Fisher Scientific) using a combination of *Clostridium histolyticum* collagenase type I (cat no: J13820.03, Thermo Fisher Scientific) (475 μg/mL) and type II (cat no: 17101015, Thermo Fisher Scientific) (125 μg/mL) at 37.4°C and 5% CO_2_. The resulting cells were centrifuged at 1300 rpm for 10 min, resuspended in DMEM with 10% fetal bovine serum (FBS; cat no: A5670801, Thermo Fisher Scientific), and incubated at 37°C with 5% CO_2_. Cells were cultured for 21 days before being used in experiments [20].

### 2.5 Application of Tocilizumab

The study involved untreated (control) and Tocilizumab‐treated groups. Tocilizumab was prepared at a concentration of 10 μg/mL by diluting a 100 mL stock solution of Actemra with isotonic saline and DMEM. The drug was applied to the primary chondrocyte cultures, and analyses were performed at 1, 7, and 15 days post‐treatment. Half maximal inhibitory concentrations (IC50) were tested by MTT (Vybrant MTT Cell Proliferation Assay Kit (V‐13154), Invitrogen) assay to detect cellular sensitivity to Tocilizumab.

### 2.6 MTT Assay for Cell Viability and Proliferation

Cell viability was assessed using the MTT assay. Cells were seeded in 96‐well plates at a density of 1.6 × 10^4^ cells per well. After treatment, 100 μL of MTT solution (12 mM) was added to each well and incubated for 2 h at 37°C. The resulting formazan crystals were dissolved using dimethyl sulfoxide (DMSO), and absorbance was measured at 540 nm to determine cell viability. The viability of control cells was set to 100%, and the percentage viability of treated groups was calculated accordingly [21].

### 2.7 AO/PI and Hoechst Staining

To evaluate membrane integrity and nuclear morphology, cells were stained with acridine orange (AO)/propidium iodide (PI) and Hoechst 33342. For AO/PI staining, cells were incubated with 5 μg/mL AO and 5 μg/mL PI in PBS for 5 min at room temperature. Live cells exhibited green fluorescence (AO‐positive), while apoptotic or necrotic cells displayed red fluorescence (PI‐positive) under a fluorescence microscope. For Hoechst staining, cells were fixed with 4% paraformaldehyde for 10 min, washed with PBS, and stained with Hoechst 33342 (1 μg/mL) for 10 min. Microscopic observations were performed using a phase contrast microscope (Leica DM2500) and an inverted microscope (Olympus CKX41) at 500× magnification. Stained cells were visualized to assess nuclear integrity and chromatin condensation, providing additional insights into potential apoptotic changes [22].

### 2.8 Western Blotting for BMP‐2, HIF‐1α, IL‐1β, SOX‐9, and IL‐6 Expression

Proteins were extracted from control and treated cells using RIPA buffer and quantified by the Bradford assay. Equal protein amounts were separated by 10% SDS‐PAGE and transferred to polyvinylidene difluoride (PVDF) membranes. Membranes were blocked with 5% nonfat milk and incubated overnight with primary antibodies against BMP‐2 (Cat no: PA5‐85956, Thermo Fisher Scientific), HIF‐1α (Cat no: MA1‐516, Thermo Fisher Scientific), IL‐1β (Cat no: MA5‐23691, Thermo Fisher Scientific), SOX‐9 (Cat no: MA5‐17177, Thermo Fisher Scientific), and β‐actin (Cat no: MA1‐140, Thermo Fisher Scientific). After washing, membranes were incubated with HRP‐conjugated secondary antibodies, and protein bands were visualized using the WesternBreeze Chemiluminescent Kit (Cat no: WB7104, Thermo Fisher Scientific). Band intensities were analyzed with ImageJ software, with β‐actin serving as the loading control [23].

### 2.9 Statistical Analysis

All experiments were conducted in triplicate, with data expressed as mean ± standard deviation (SD). Statistical significance was determined using one‐way analysis of variance (ANOVA) followed by Tukey's post hoc test. Statistical analyses were performed using R software (v4.3.3), and *p*‐values less than 0.05 were considered statistically significant.

## 3 Results

In this study, we investigated the regulatory effects of Tocilizumab on key molecular pathways in human primary chondrocytes, focusing on the roles of BMP‐2, HIF‐1α, IL‐1β, SOX‐9, and IL‐6. Utilizing a combination of bioinformatic and in vitro analyses, we explored the involvement of these genes in inflammatory signaling, differentiation processes, and cellular viability. Reactome and GO enrichment analyses revealed significant pathways and GO terms associated with inflammation and chondrocyte functionality. Additionally, PPI network analysis highlighted the interconnected roles of these genes within cellular signaling networks. Complementary experiments, including Western blot and MTT assays and staining analyses, provided further insight into the dynamic expression of these proteins over time and the cytostatic effects of Tocilizumab on chondrocytes. Together, these findings underscore the multifaceted impact of Tocilizumab on chondrocyte biology, with implications for cartilage health and inflammation modulation.

**FIGURE 1:**
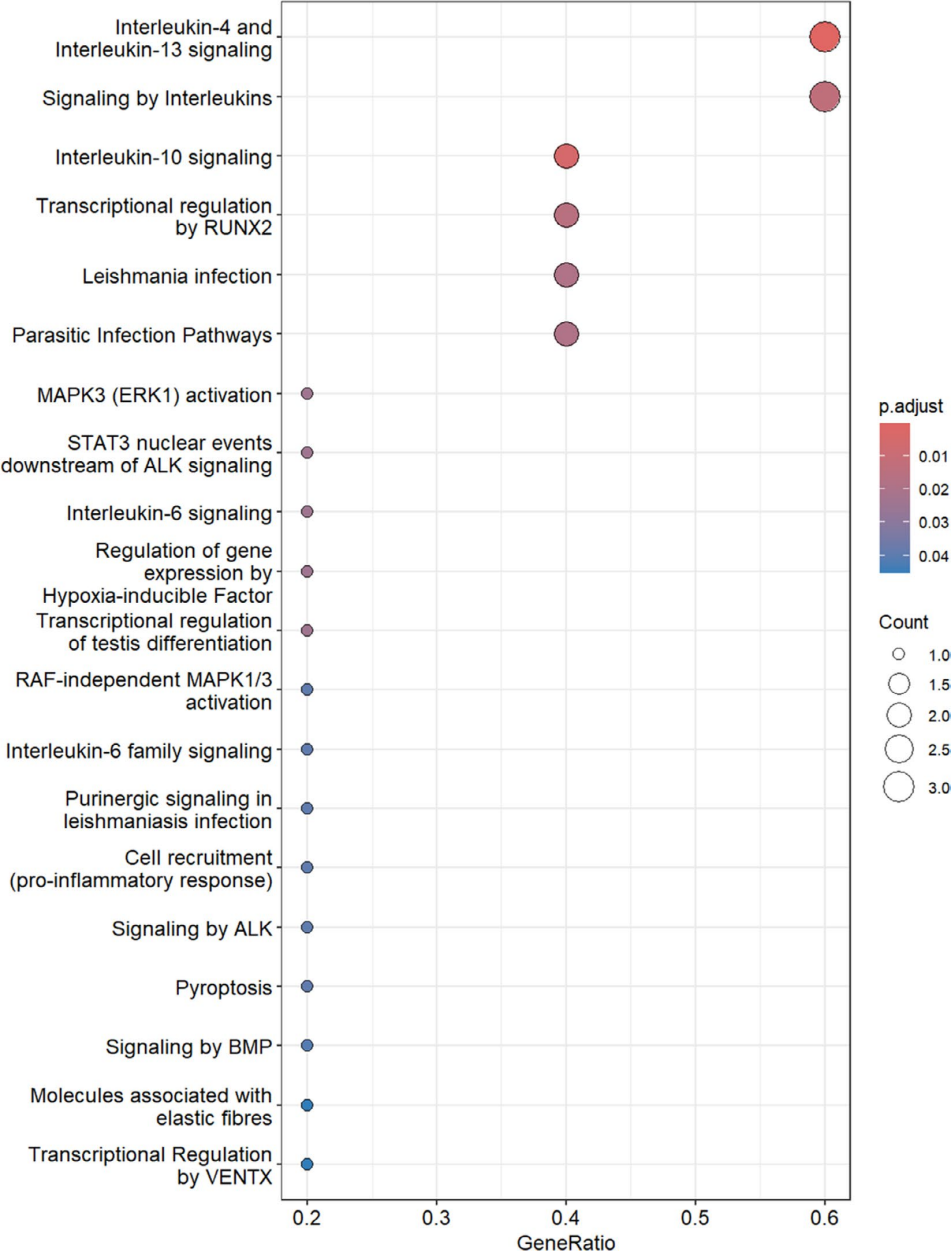
Reactome pathway enrichment analysis of BMP‐2, HIF‐1α, IL‐1β, SOX‐9, and IL‐6.

The Reactome pathway enrichment analysis revealed several significant pathways associated with the BMP‐2, HIF‐1α, IL‐6, and SOX‐9 genes in human primary chondrocytes treated with Tocilizumab (Figure 1). Among the most significantly enriched pathways were interleukin‐4 and interleukin‐13 signaling, Transcriptional regulation by Runt‐related transcription factor 2 (RUNX2), and signaling by interleukins. These pathways are highly relevant to immune regulation and inflammation [24, 25, 26], suggesting that Tocilizumab may influence chondrocyte function through the modulation of cytokine signaling and transcriptional regulation.

Interleukin‐4 and interleukin‐13 signaling were the most significantly enriched pathways, highlighting the potential role of these cytokines in the inflammatory responses of chondrocytes [25]. RUNX2, a key transcription factor involved in bone and cartilage development [27], also appeared prominently in the analysis, indicating that Tocilizumab may influence cartilage differentiation and repair processes. Additionally, pathways such as MAPK3 (ERK1) activation and (IL‐6) signaling were also enriched, reflecting the involvement of these signaling cascades in cellular responses to inflammation and tissue stress [28, 29]. Overall, the results suggest that Tocilizumab may exert its therapeutic effects on human chondrocytes by modulating both inflammatory and transcriptional regulation pathways, with potential impacts on cartilage maintenance and repair [23, 30].

**FIGURE 2:**
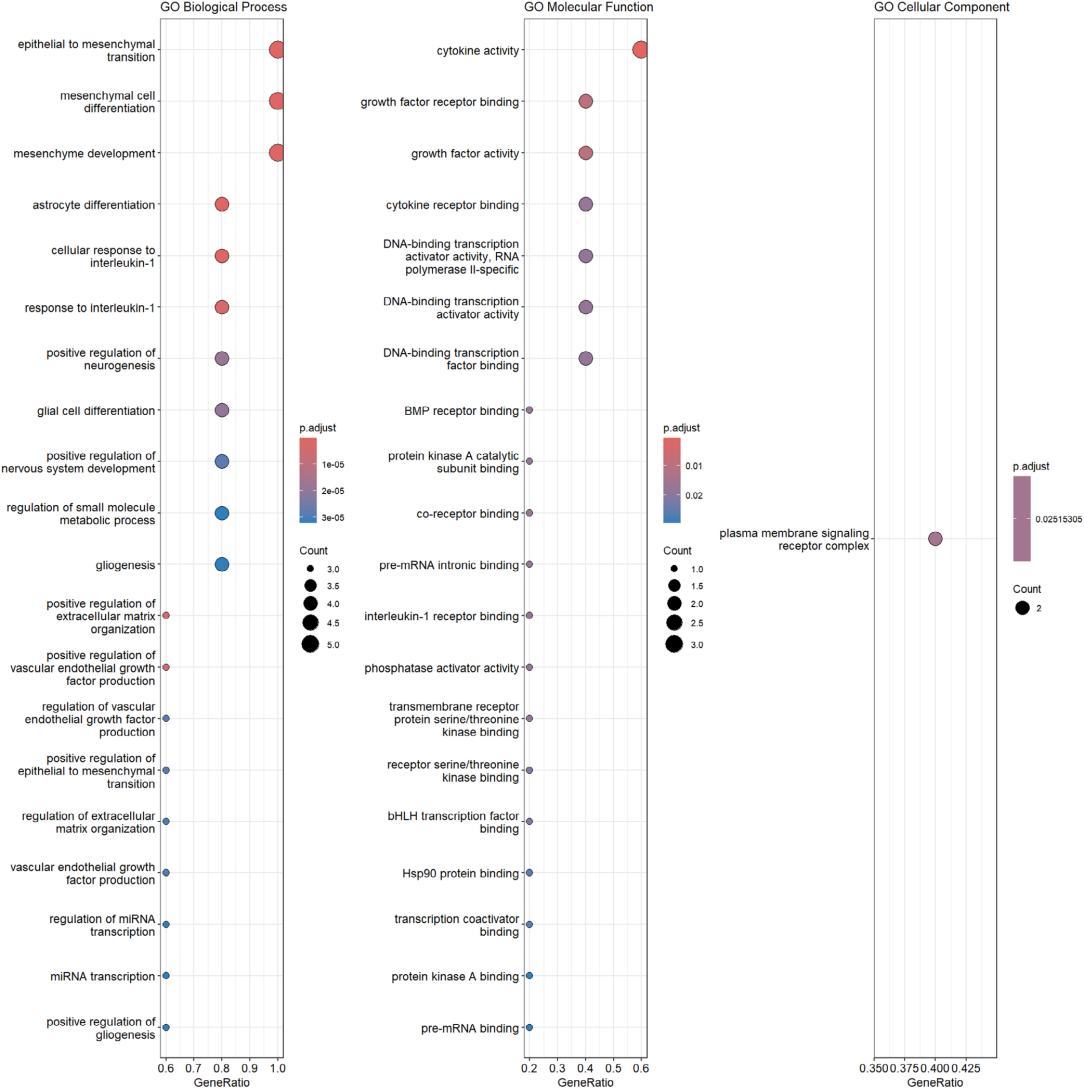
GO enrichment analysis of BMP‐2, HIF‐1α, IL‐1β, SOX‐9, and IL‐6 genes. The analysis includes three categories: biological process, molecular function, and cellular component.

The GO enrichment analysis revealed significant BPs, MFs, and CCs associated with the BMP‐2, HIF‐1α, IL‐6, and SOX‐9 genes in human primary chondrocytes treated with Tocilizumab (Figure 2). In the BP category, key enriched terms included epithelial to mesenchymal transition, mesenchymal cell differentiation, and regulation of extracellular matrix organization, all of which are crucial in tissue remodeling and repair mechanisms. The high enrichment of epithelial to mesenchymal transition [31] and mesenchymal cell differentiation suggests that these processes may play a significant role in the chondrocyte response to Tocilizumab treatment. In the MF category, important enriched terms such as growth factor activity, cytokine activity, and BMP receptor binding were identified. These MFs are closely related to the regulation of cellular communication, inflammation, and growth responses, which are critical for maintaining chondrocyte function and cartilage integrity. For the CC category, the most significantly enriched term was the plasma membrane signaling receptor complex. These results highlight the potential of Tocilizumab to modulate both inflammatory responses and cellular differentiation processes in human primary chondrocytes, suggesting that the drug may exert its effects through pathways related to tissue repair, inflammation, and extracellular matrix organization [8, 32].

**FIGURE 3:**
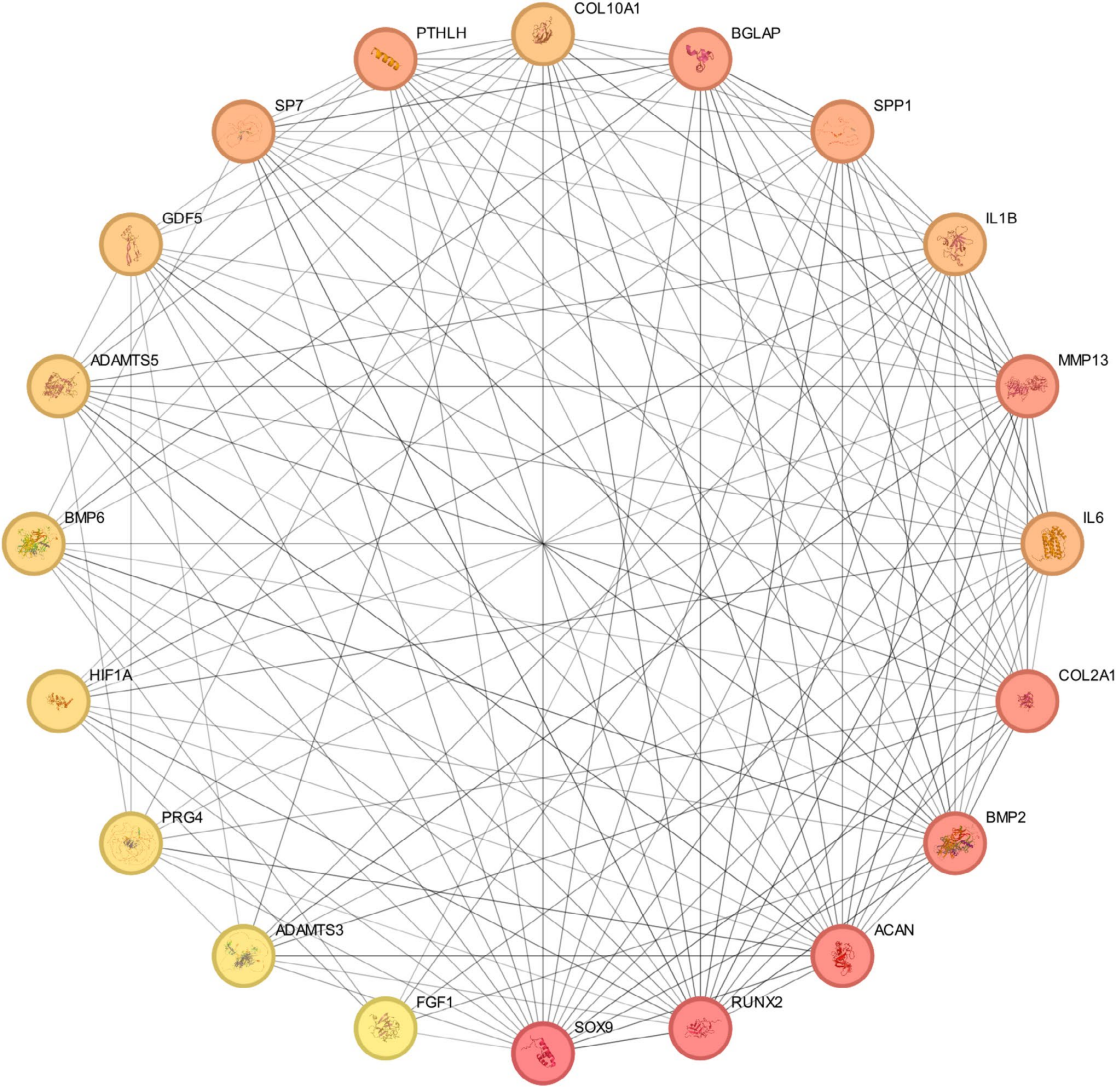
PPI network analysis of BMP‐2, HIF‐1α, IL‐1β, SOX‐9, and IL‐6. The network displays high‐confidence interactions between proteins involved in key biological processes such as cartilage development, extracellular matrix organization, and inflammation.

The PPI network analysis identified a dense interaction network involving the BMP‐2, HIF‐1α, IL‐1β, SOX‐9, and IL‐6 genes, along with several key interacting proteins in human primary chondrocytes treated with Tocilizumab (Figure 3). Notably, the network includes numerous high‐confidence interactions, with a focus on pathways related to cartilage development, inflammation, and matrix organization. Key proteins such as RUNX2, MMP13, COL2A1, ACAN, and BMP‐2 were identified as central nodes, indicating their crucial roles in maintaining cartilage integrity and regulating inflammatory responses [24]. Additionally, HIF‐1α, a major regulator of hypoxic responses, and IL‐6, a key inflammatory mediator, were identified as significant hubs in the network, reflecting their central roles in both cellular stress responses and inflammation modulation [10]. The analysis also highlighted several proteins involved in matrix remodeling, including A Disintegrin and Metalloproteinase with Thrombospondin motifs (ADAMTS) and MMP13, which are known to be involved in extracellular matrix breakdown and cartilage degradation [33, 34, 35], suggesting that Tocilizumab may influence matrix homeostasis in chondrocytes. Overall, the STRING PPI network suggests that Tocilizumab may exert its effects on chondrocytes by modulating key proteins involved in cartilage development, extracellular matrix organization, and inflammatory signaling pathways, which are essential for maintaining cartilage health and reducing degeneration.

**FIGURE 4:**
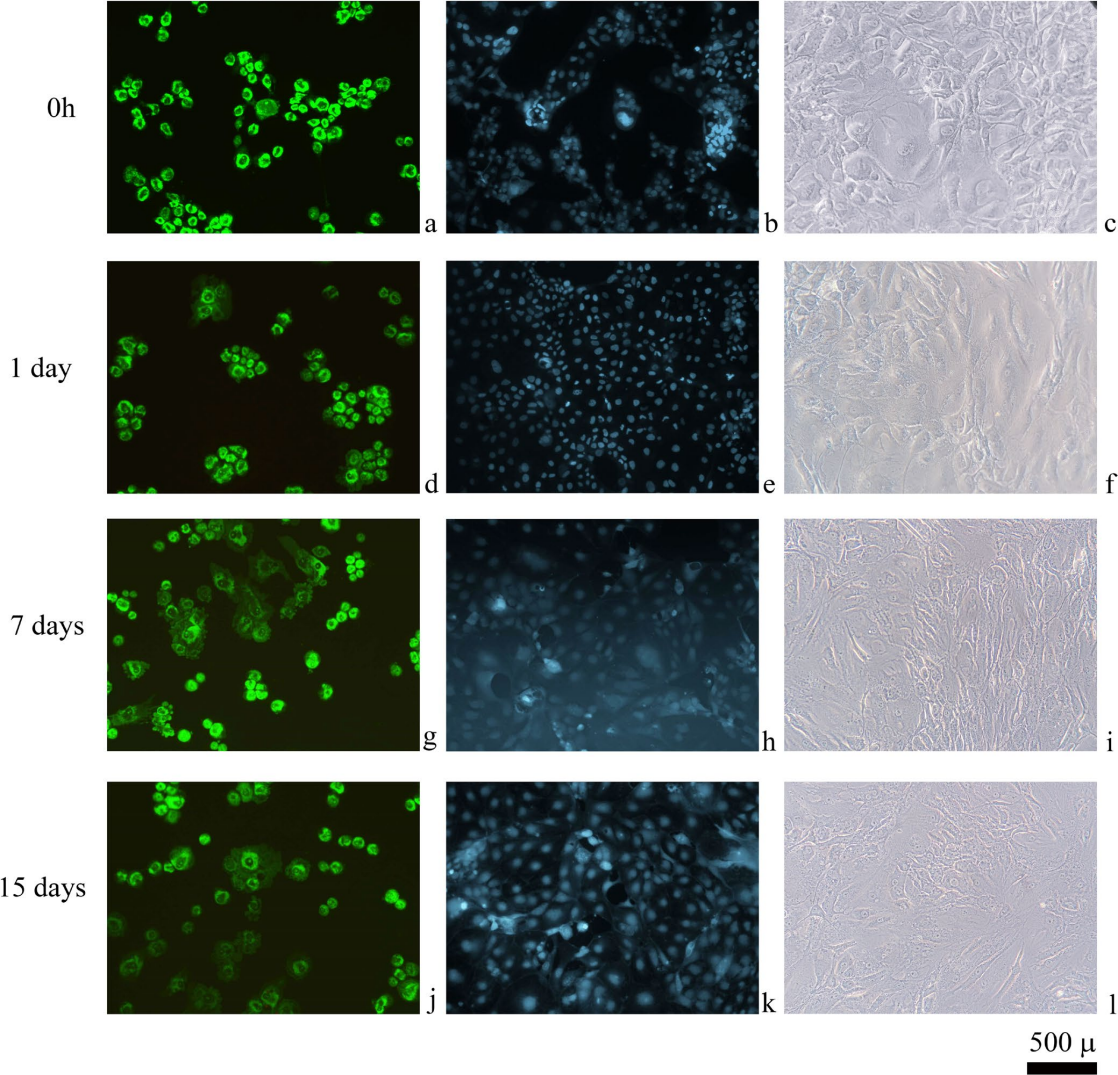
Microscopic staining of chondrocytes with AO/PI and Hoechst stains, first lane (a–c) control group samples, (d–f) micrographs of Tocilizumab‐applied cultures for 1 day, (g–i) 7 days, and (j–l) 15 days. First column: acridine orange/propidium iodide‐stained cultures (20× magnification), second column: Hoechst‐stained cultures (20× magnification). Third column: inverted microscopy images.

Microscopic staining results are shown in Figure 4, presenting the viability and nuclear morphology of chondrocytes treated with Tocilizumab (Figure 4). AO/PI staining reveals predominantly green fluorescence, indicating a high proportion of viable chondrocytes across both control and Tocilizumab‐treated groups. The lack of red fluorescence (propidium iodide) suggests minimal cell death or membrane compromise, even with extended exposure to Tocilizumab. Similarly, Hoechst staining results highlight intact nuclear morphology in both control and treated groups. The nuclei appear uniformly stained and well defined, with no evidence of chromatin condensation or nuclear fragmentation typically associated with apoptosis. These results suggest that Tocilizumab does not induce significant cytotoxic effects or nuclear alterations in primary human chondrocytes under the conditions tested [23].

**FIGURE 5:**
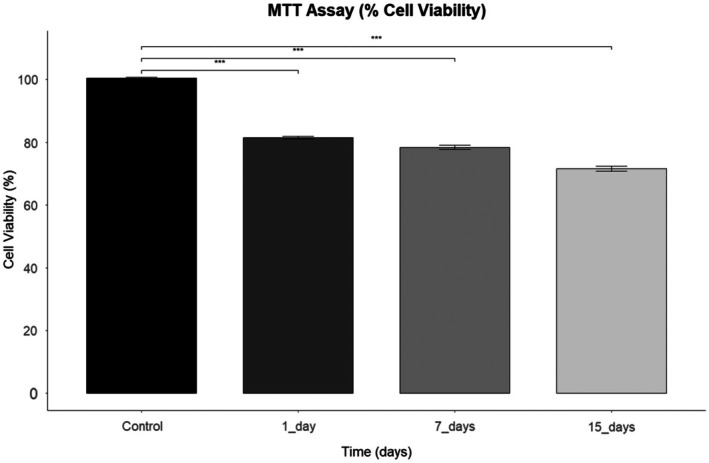
MTT assay showing the percentage cell viability of human primary chondrocytes treated with Tocilizumab at different time points (Control, 1, 7, 15 days). Error bars represent standard deviation across replicates. ***Data represent mean ± SD of at least three independent experiments. Statistical significance was determined using one‐way ANOVA followed by post hoc analysis (*p* < 0.05 considered significant).

The effects of Tocilizumab on cell viability in human primary chondrocytes were evaluated using MTT analysis at different time points. The control group exhibited the highest cell viability, whereas the 15‐day treatment group showed the lowest viability levels. Statistical evaluation of the MTT assay results revealed a significant, time‐dependent reduction in chondrocyte viability after Tocilizumab treatment (*p* < 0.005) (Figure 5). These findings suggest that changes in cell viability may reflect the potential impact of the drug on chondrocyte physiology [23].

**FIGURE 6:**
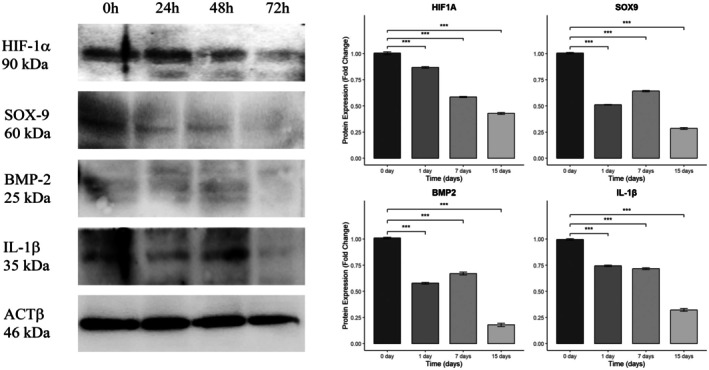
Western blot analysis of HIF‐1α, SOX‐9, BMP‐2, and IL‐1β protein expression levels in human primary chondrocytes treated with Tocilizumab across different time points. Representative Western blot images showing the expression levels of HIF‐1α, SOX‐9, BMP‐2, and IL‐1β in human primary chondrocytes following Tocilizumab treatment at 0, 1, 7, and 15 days. Protein bands were quantified by densitometric analysis and normalized to β‐actin. Data are representative of at least three independent experiments. ***Data are expressed as mean ± SD from replicate experiments.

Western blot analysis examined the expression of HIF‐1α, SOX‐9, BMP‐2, and IL‐1β proteins in chondrocytes exposed to Tocilizumab treatment. A notable downward trend was observed in SOX‐9 and BMP‐2 protein levels by day 15, while HIF‐1α and IL‐1β showed a more moderate decrease over the treatment period (Figure 6). Compared to the control (0 day), a statistically significant time‐dependent decrease in protein expression was observed at 1, 7, and 15 days following Tocilizumab treatment (*p* < 0.005) [23].

## 4 Discussion

In this study, the effects of Tocilizumab on human primary chondrocyte cells were evaluated through BMP‐2, HIF‐1α, IL‐1β, SOX‐9, and IL‐6 genes. The Reactome and GO enrichment analyses indicated significant involvement of pathways such as interleukin signaling, BMP signaling, and mitogen‐activated protein kinase (MAPK) pathways in chondrocyte regulation. Specifically, Reactome pathways like “Interleukin‐6 signaling” and “MAPK3 (ERK1) activation” are central to inflammation and cellular response regulation. Given Tocilizumab's inhibitory effects on IL‐6 signaling, it may influence metabolic processes and differentiation pathways critical to chondrocyte health and function.

The STRING PPI network further highlighted the interaction between BMP‐2, HIF‐1α, IL‐1β, SOX‐9, and IL‐6, alongside additional proteins associated with inflammation and developmental processes, such as RUNX2 and tenomodulin (TNMD). This dense interaction network underscores the interconnected roles of these genes, with potential downstream effects on chondrocyte differentiation and function. In particular, BMP‐2 and SOX‐9 play crucial roles in chondrogenesis and differentiation, while HIF‐1α is vital for cellular adaptation under low oxygen conditions. HIF‐1α has also been shown to transcriptionally regulate SOX‐9 expression during hypoxic stress, thereby linking hypoxia response to chondrocyte differentiation processes [36]. The observed reduction in SOX‐9 and HIF‐1α expression over time suggests that Tocilizumab may impact the differentiation and oxygen sensitivity of chondrocytes, especially under prolonged inflammatory conditions [36].

In the Western blot analysis, an early increase in BMP‐2 and IL‐1β protein expression was observed, which may be related to the initial activation of inflammatory pathways. The subsequent reduction in BMP‐2 expression could be interpreted as a compensatory feedback mechanism to limit excessive matrix remodeling, as sustained BMP‐2 activation can potentially promote chondrocyte hypertrophy [9]. However, the subsequent decrease in HIF‐1α and SOX‐9 protein levels over time indicates that Tocilizumab could suppress regulatory mechanisms essential to chondrocyte differentiation and cellular adaptation in the long term. This reduction suggests a potential decrease in the cells' ability to adapt within an anti‐inflammatory environment, impacting their functionality and differentiation capabilities [36].

The MTT cell viability assay results demonstrated a time‐dependent decline in cell viability, potentially reflecting the cytostatic effects of Tocilizumab on chondrocytes. IL‐6 has been reported to inhibit chondrocyte proliferation [15]; however, in our study, the blockade of IL‐6 signaling by Tocilizumab was associated with a time‐dependent reduction in chondrocyte viability, indicating that additional regulatory mechanisms may be involved in the cytostatic effects observed. This reduction in viability may arise from the drug's inhibitory effects on inflammatory signaling pathways, which could subsequently decrease chondrocyte growth and proliferation capacity. The response of chondrocytes to prolonged Tocilizumab exposure highlights essential findings regarding cartilage integrity and viability, particularly in treating chronic inflammatory conditions.

In the AO/PI and Hoechst staining analyses, chondrocytes treated with Tocilizumab exhibited consistent green fluorescence and intact nuclear morphology, with no observable increase in red‐stained (nonviable) cells or signs of nuclear deformation. This outcome suggests that while Tocilizumab may have a cytostatic effect, as indicated by reduced proliferation in MTT assays, it does not compromise membrane integrity or induce apoptosis within the timeframe assessed. These findings emphasize Tocilizumab's favorable safety profile concerning cellular integrity in chondrocytes, even though prolonged treatment could affect cell viability and proliferation. These findings emphasize that Tocilizumab preserves cellular and nuclear integrity under the tested conditions, although prolonged treatment may impact overall cell viability and proliferation capacity.

Overall, the findings of this study demonstrate that Tocilizumab significantly modulates pathways influencing inflammatory and differentiation processes in human primary chondrocytes. The inhibition of IL‐6 signaling may indirectly impact cartilage synthesis and repair mechanisms and could lead to a gradual reduction in cellular viability over time. These results provide a valuable foundation for future studies to elucidate the long‐term effects of Tocilizumab on cartilage tissue in chronic inflammatory diseases and to assess its therapeutic potential in cartilage preservation and regeneration.

## Author Contributions

Bugrahan Regaip Kilinc: conceptualization, bioinformatics analysis, data curation, in silico and in vitro experiments, writing – original draft. Feyza Kostak: methodology, data analysis. Omer Faruk Yilmaz: methodology, collecting osteochondral tissue. Suray Pehlivanoglu: methodology, data analysis, writing – original draft. Duygu Yasar Sirin: methodology, data analysis, supervision, writing – review and editing. All authors read and approved the final manuscript.

## Ethics Statement

This study was conducted in accordance with the ethical standards of the (TNKÜ Girişimsel Olmayan Klinik Araştırmalar Etik Kurulu) and was approved under Ethics Approval Number: 2024.302.11.09.

## Consent

Written informed consent was obtained from all donors prior to cartilage tissue collection.

## Conflicts of Interest

The authors declare no conflicts of interest.

## Data Availability

The data that support the findings of this study are available from the corresponding author upon reasonable request.

## References

[1] T. Pap and A. Korb‐Pap , “Cartilage Damage in Osteoarthritis and Rheumatoid Arthritis—Two Unequal Siblings,” Nature Reviews Rheumatology 11, no. 10 (2015): 606–615, 10.1038/NRRHEUM.2015.95. 26195338

[2] H. Muir , “The Chondrocyte, Architect of Cartilage. Biomechanics, Structure, Function and Molecular Biology of Cartilage Matrix Macromolecules,” BioEssays 17, no. 12 (1995): 1039–1048, 10.1002/BIES.950171208. 8634065

[3] S. R. Goldring and M. B. Goldring , “The Role of Cytokines in Cartilage Matrix Degeneration in Osteoarthritis,” Clinical Orthopaedics and Related Research 427 (2004): S27–S36, 10.1097/01.BLO.0000144854.66565.8F. 15480070

[4] A. J. Schuerwegh , E. J. Dombrecht , W. J. Stevens , J. F. Van Offel , C. H. Bridts , and L. S. De Clerck , “Influence of Pro‐Inflammatory (IL‐1α, IL‐6, TNF‐α, IFN‐γ) and Anti‐Inflammatory (IL‐4) Cytokines on Chondrocyte Function,” Osteoarthritis and Cartilage 11, no. 9 (2003): 681–687, 10.1016/S1063-4584(03)00156-0. 12954239

[5] R. Wiegertjes , F. A. J. Van De Loo , and E. N. Blaney Davidson , “A Roadmap to Target Interleukin‐6 in Osteoarthritis,” Rheumatology (Oxford) 59, no. 10 (2020): 2681–2694, 10.1093/RHEUMATOLOGY/KEAA248. 32691066 PMC7516110

[6] Q. Yao , X. Wu , C. Tao , et al., “Osteoarthritis: Pathogenic Signaling Pathways and Therapeutic Targets,” Signal Transduction and Targeted Therapy 8, no. 1 (2023): 1–31, 10.1038/s41392-023-01330-w. 36737426 PMC9898571

[7] M. Mihara , Y. Ohsugi , and T. Kishimoto , “Tocilizumab, a Humanized Anti‐Interleukin‐6 Receptor Antibody, for Treatment of Rheumatoid Arthritis,” Open Access Rheumatology: Research and Reviews 3 (2011): 19–29, 10.2147/OARRR.S17118. 27790001 PMC5074778

[8] M. Hashizume , S. L. Tan , J. Takano , et al., “Tocilizumab, a Humanized Anti‐IL‐6R Antibody, as an Emerging Therapeutic Option for Rheumatoid Arthritis: Molecular and Cellular Mechanistic Insights,” International Reviews of Immunology 34, no. 3 (2015): 265–279, 10.3109/08830185.2014.938325. 25099958

[9] Z. H. Deng , Y. S. Li , X. Gao , G. H. Lei , and J. Huard , “Bone Morphogenetic Proteins for Articular Cartilage Regeneration,” Osteoarthritis and Cartilage 26, no. 9 (2018): 1153–1161, 10.1016/J.JOCA.2018.03.007. 29580979

[10] C. Y. Zeng , X. F. Wang , and F. Z. Hua , “HIF‐1α in Osteoarthritis: From Pathogenesis to Therapeutic Implications,” Frontiers in Pharmacology 13 (2022): 927126, 10.3389/FPHAR.2022.927126. 35865944 PMC9294386

[11] H. Song and K. H. Park , “Regulation and Function of SOX9 During Cartilage Development and Regeneration,” Seminars in Cancer Biology 67 (2020): 12–23, 10.1016/J.SEMCANCER.2020.04.008. 32380234

[12] K. Hino , A. Saito , M. Kido , et al., “Master Regulator for Chondrogenesis, Sox9, Regulates Transcriptional Activation of the Endoplasmic Reticulum Stress Transducer BBF2H7/CREB3L2 in Chondrocytes,” Journal of Biological Chemistry 289, no. 20 (2014): 13810–13820, 10.1074/JBC.M113.543322. 24711445 PMC4022855

[13] P. Kongdang , C. Chokchaitaweesuk , S. Tangyuenyong , and S. Ongchai , “Proinflammatory Effects of IL‐1β Combined With IL‐17A Promoted Cartilage Degradation and Suppressed Genes Associated With Cartilage Matrix Synthesis In Vitro,” Molecules 24, no. 20 (2019): 3682, 10.3390/MOLECULES24203682. 31614911 PMC6833041

[14] C. R. Flannery , C. B. Little , C. E. Hughes , C. L. Curtis , B. Caterson , and S. A. Jones , “IL‐6 and Its Soluble Receptor Augment Aggrecanase‐Mediated Proteoglycan Catabolism in Articular Cartilage,” Matrix Biology 19, no. 6 (2000): 549–553, 10.1016/S0945-053X(00)00111-6. 11068209

[15] A. Jikko , T. Wakisaka , M. Iwamoto , et al., “Effects of INTERLEUKIN‐6 on Proliferation and Proteoglycan Metabolism in Articular Chondrocyte Cultures,” Cell Biology International 22, no. 9–10 (1998): 615–621, 10.1006/CBIR.1998.0304. 10452831

[16] G. Yu , L. G. Wang , Y. Han , and Q. Y. He , “ClusterProfiler: An R Package for Comparing Biological Themes Among Gene Clusters,” OMICS: A Journal of Integrative Biology 16, no. 5 (2012): 284–287, 10.1089/OMI.2011.0118. 22455463 PMC3339379

[17] G. Yu and Q. Y. He , “ReactomePA: An R/Bioconductor Package for Reactome Pathway Analysis and Visualization,” Molecular BioSystems 12, no. 2 (2016): 477–479, 10.1039/C5MB00663E. 26661513

[18] C. von Mering , M. Huynen , D. Jaeggi , S. Schmidt , P. Bork , and B. Snel , “STRING: A Database of Predicted Functional Associations Between Proteins,” Nucleic Acids Research 31, no. 1 (2003): 258–261, 10.1093/NAR/GKG034. 12519996 PMC165481

[19] C. H. Chin , S. H. Chen , H. H. Wu , C. W. Ho , M. T. Ko , and C. Y. Lin , “cytoHubba: Identifying Hub Objects and Sub‐Networks From Complex Interactome,” BMC Systems Biology 8, no. Suppl 4 (2014): S11, 10.1186/1752-0509-8-S4-S11. 25521941 PMC4290687

[20] M. Isyar , I. Yilmaz , D. Yasar Sirin , S. Yalcin , O. Guler , and M. Mahirogullari , “A Practical Way to Prepare Primer Human Chondrocyte Culture,” Journal of Orthopaedics 13, no. 3 (2016): 162–167, 10.1016/J.JOR.2016.03.008. 27408489 PMC4919283

[21] P. Kumar , A. Nagarajan , and P. D. Uchil , “Analysis of Cell Viability by the MTT Assay,” Cold Spring Harbor Protocols 2018, no. 6 (2018): 469–471, 10.1101/PDB.PROT095505. 29858338

[22] C. Lema , A. Varela‐Ramirez , and R. J. Aguilera , “Differential Nuclear Staining Assay for High‐Throughput Screening to Identify Cytotoxic Compounds,” Current Cell Biology 1, no. 1 (2011): 1, https://pmc.ncbi.nlm.nih.gov/articles/PMC4816492/. PMC4816492 27042697

[23] I. Yilmaz , H. Akalan , D. Y. Sirin , et al., “Effects of Tocilizumab on Intervertebral Disc Degeneration, Cell Senescence and Inflammation via BMP‐2, Hif‐1α, IL‐1β and SOX9,” International Journal of Pharmacology 19, no. 6 (2023): 825–833, 10.3923/IJP.2023.825.833.

[24] M. Lv , Y. Zhou , S. W. Polson , et al., “Identification of Chondrocyte Genes and Signaling Pathways in Response to Acute Joint Inflammation,” Scientific Reports 9, no. 1 (2019): 93, 10.1038/s41598-018-36500-2. 30643177 PMC6331554

[25] M. Iwaszko , S. Biały , and K. Bogunia‐Kubik , “Significance of Interleukin (IL)‐4 and IL‐13 in Inflammatory Arthritis,” Cells 10 (2021): 3000, 10.3390/CELLS10113000. 34831223 PMC8616130

[26] S. Wahlen , F. Matthijssens , W. van Loocke , et al., “The Transcription Factor RUNX2 Drives the Generation of Human NK Cells and Promotes Tissue Residency,” eLife 11 (2022): e80320, 10.7554/ELIFE.80320. 35793229 PMC9259014

[27] D. Chen , D. J. Kim , J. Shen , Z. Zou , and R. J. O'Keefe , “Runx2 Plays a Central Role in Osteoarthritis Development,” Journal of Orthopaedic Translation 23 (2020): 132–139, 10.1016/J.JOT.2019.11.008. 32913706 PMC7452174

[28] T. Tanaka , M. Narazaki , and T. Kishimoto , “IL‐6 in Inflammation, Immunity, and Disease,” Cold Spring Harbor Perspectives in Biology 6, no. 10 (2014): a016295, 10.1101/CSHPERSPECT.A016295. 25190079 PMC4176007

[29] J. M. Kyriakis and J. Avruch , “Mammalian MAPK Signal Transduction Pathways Activated by Stress and Inflammation: A 10‐Year Update,” Physiological Reviews 92, no. 2 (2012): 689–737, 10.1152/PHYSREV.00028.2011. 22535895

[30] A. Rubbert‐Roth , D. E. Furst , J. M. Nebesky , A. Jin , and E. Berber , “A Review of Recent Advances Using Tocilizumab in the Treatment of Rheumatic Diseases,” Rheumatology and Therapy 5, no. 1 (2018): 21–42, 10.1007/S40744-018-0102-X. 29502236 PMC5935615

[31] J. P. Thiery , H. Acloque , R. Y. J. Huang , and M. A. Nieto , “Epithelial‐Mesenchymal Transitions in Development and Disease,” Cell 139, no. 5 (2009): 871–890, 10.1016/J.CELL.2009.11.007. 19945376

[32] M. Shimasaki , S. Ueda , M. Sakurai , N. Kawahara , Y. Ueda , and T. Ichiseki , “Celecoxib Combined With Tocilizumab has Anti‐Inflammatory Effects and Promotes the Recovery of Damaged Cartilage via the Nrf2/HO‐1 Pathway In Vitro,” Biomolecules 14, no. 12 (2024): 1636, 10.3390/BIOM14121636. 39766343 PMC11727524

[33] T. Li , J. Peng , Q. Li , Y. Shu , P. Zhu , and L. Hao , “The Mechanism and Role of ADAMTS Protein Family in Osteoarthritis,” Biomolecules 12 (2022): 959, 10.3390/BIOM12070959. 35883515 PMC9313267

[34] R. H. Song , M. D. Tortorella , A. M. Malfait , et al., “Aggrecan Degradation in Human Articular Cartilage Explants Is Mediated by Both ADAMTS‐4 and ADAMTS‐5,” Arthritis and Rheumatism 56, no. 2 (2007): 575–585, 10.1002/ART.22334. 17265492

[35] Q. Hu and M. Ecker , “Overview of MMP‐13 as a Promising Target for the Treatment of Osteoarthritis,” International Journal of Molecular Sciences 22, no. 4 (2021): 1742, 10.3390/IJMS22041742. 33572320 PMC7916132

[36] R. Amarilio , S. V. Viukov , A. Sharir , I. Eshkar‐Oren , R. S. Johnson , and E. Zelzer , “HIF1alpha Regulation of Sox9 Is Necessary to Maintain Differentiation of Hypoxic Prechondrogenic Cells During Early Skeletogenesis,” Development (Cambridge, England) 134, no. 21 (2007): 3917–3928, 10.1242/DEV.008441. 17913788

